# Transparent Conductive Films Based on rGO/AgNW/PET for Electrical Heating and Electromagnetic Interference Shielding Applications

**DOI:** 10.3390/nano16110655

**Published:** 2026-05-24

**Authors:** Ke Hu, Wen-Hao Geng, Hong-Zhang Geng

**Affiliations:** 1 Tianjin Key Laboratory of Advanced Fibers and Energy Storage, School of Material Science and Engineering, Tiangong University, Tianjin 300387, China; 2331020410@tiangong.edu.cn; 2 Tianji Zhencai Technology (Hebei) Co., Ltd., Cangzhou 061000, China; tjzc001@outlook.com; 3 Hebei Province Technology R&D Platform, Hebei Industrial Technology Research Institute of Membranes, Cangzhou Institute of Tiangong University, Cangzhou 061000, China

**Keywords:** reduced graphene oxide, TCFs, AgNWs, electrical heating, EMI

## Abstract

Flexible transparent conductive films (TCFs) and their applications have attracted extensive interest. Silver nanowires (AgNWs) have been explored to replace conventional indium tin oxide (ITO) due to their high optical transmittance and superior electrical conductivity. Nevertheless, AgNWs tend to oxidize under ambient conditions, which weakens the conductive network and limits long-term performance. Spraying reduced graphene oxide (rGO) can stabilize the conductive network and inhibit oxidation, thereby enhancing the overall properties of the films. In this work, rGO/AgNW/PET TCFs were prepared using a spray-coating approach. The transmittance of the rGO/AgNW/PET TCFs was measured at 77% at 550 nm, accompanied by a sheet resistance of 6.8 Ω/sq. The films achieved the surface temperature of 95 °C at 6 V with stable operation while also achieving an electromagnetic interference shielding effectiveness of 27 dB. This structural design improves both performance and stability, offering great potential for flexible TCFs in advanced optoelectronic applications.

## 1. Introduction

Flexible transparent conductive films (TCFs) serve as essential functional elements in a variety of optoelectronic devices [1,2,3]. Examples of their applications include systems for electromagnetic interference (EMI) shielding, transparent flexible heating elements, and solar energy cells [4,5,6,7]. Indium tin oxide (ITO), known for its excellent performance, has long been the dominant material in the field of TCFs [8,9,10]. However, the limited availability and high cost of indium, together with the intrinsic brittleness of ITO films, significantly restrict their application [11]. Recently, silver nanowires (AgNWs) have shown promise as transparent conductive materials due to their excellent characteristics. Praveen et al. [12] prepared AgNWs that had diameters ranging from about 50 to 80 nm and lengths between 5 and 30 μm. Xu et al. [13] synthesized long AgNWs via a modified polyol method. Andrés et al. [14] achieved the rapid formation of ultralong AgNWs via a one-pot polyol method, with lengths reaching 195 μm in less than 1 h. Qian et al. [15] synthesized high-aspect-ratio AgNWs through polyol process.

Graphene oxide (GO) is a material with a two-dimensional layered structure that has atomic thickness [16,17,18,19]. However, the oxidation process disrupts the intrinsic sp^2^ conjugated network of GO, which is difficult to fully restore, thereby significantly deteriorating its electrical conductivity [20,21,22]. Hydrazine hydrate, sodium borohydride, and hydroiodic acid are among the commonly employed chemical agents for reducing GO [23,24]. Consequently, the development of environmentally friendly reduction strategies for producing reduced graphene oxide (rGO) is of considerable importance. Ascorbic acid (AA), an environmentally friendly reductant, efficiently removes oxygen-containing groups from GO under mild conditions, partially restoring the graphene network while avoiding the toxicity of conventional strong reducing agents [25,26].

Bai et al. [27] reported Mxene–AgNW TCFs with a sheet resistance of 16 Ω/sq, a transmittance of 86.1%, and an EMI shielding effectiveness (EMI SE) of 27.8 dB. Liu et al. [28] developed PET/AgNW/Ti_3_C_2_T_x_/PVA–PSS films with a transmittance of 81% and an EMI SE of 30.5 dB. Yan et al. [29] designed graphene/AgNW/graphene TCFs, achieving a low sheet resistance below 15 Ω/sq, a high transmittance of 88%, and an EMI SE of 31 dB. However, these studies mainly focused on enhancing EMI shielding performance, with limited attention to electrothermal properties and multifunctional integration, which restricts their applications in flexible multifunctional electronic devices. In addition, Zhang et al. [30] reported an AgNW/PEDOT: PSS@Ni electrode with a sheet resistance of 29 Ω/sq, a transmittance of 78.18%, and an EMI SE of 19.64 dB. However, its EMI SE remains below 20 dB, which limits its applicability in high-performance EMI shielding applications.

In this work, a TCF based on an rGO/AgNW composite structure was developed. The study aimed to construct a conductive network with integrated high optical transparency, low sheet resistance, stable electrothermal performance, and effective EMI shielding capability. Firstly, AgNWs were obtained using a polyol method. The obtained AgNWs were then uniformly deposited onto a polyethylene terephthalate (PET) substrate through a spray-coating process to form AgNW/PET films. Subsequently, GO was reduced to rGO using AA. The rGO layer was further spray-coated onto the surface of AgNW/PET, resulting in rGO/AgNW/PET TCFs. The rGO layer protects AgNWs from oxidation and improves the stability of rGO/AgNW/PET TCFs. The transmittance at 550 nm for the rGO/AgNW/PET TCF was 77%, accompanied by a sheet resistance of 6.8 Ω/sq, demonstrating excellent optoelectronic performance. The films exhibited outstanding electrothermal performance, achieving approximately 95 °C at 6 V with stable operation. In addition, the films demonstrated an EMI SE of 27 dB.

## 2. Materials and Methods

### 2.1. Materials

GO dispersion with a thickness of approximately 0.6–1.0 nm and lateral dimensions of 0.5–5 μm was supplied by Shenzhen Suiheng Graphene Technology Co., Ltd. (Shenzhen, China). Silver nitrate (AgNO_3_) was purchased from Tianjin Yingda Rare Chemical Reagents Factory (Tianjin, China). Polyvinylpyrrolidone (PVP, M_W_ = 58,000 and 360,000) and AA were purchased from Macklin Biochemical Co., Ltd. (Shanghai, China). Ethylene glycol (EG) was provided by Kermel (Tianjin, China) Chemical Technology Co., Ltd. (Tianjin, China). Acetone was acquired from Tianjin Fengchuan Chemical Reagent Technology Co., Ltd. (Tianjin, China). PET was supplied by Tianjin Wanhua Co., Ltd. (Tianjin, China).

### 2.2. Preparation of AgNWs

Firstly, PVP with two different molecular weights (M_W_ = 58,000 and 360,000) was dissolved in EG while stirring. After the PVP was nearly completely dissolved, AgNO_3_ was added. The solution was then stirred, followed by the addition of FeCl_3_, and stirring continued. Subsequently, the stirring was stopped, and the solution was transferred into a preheated three-neck flask. The reaction system was maintained under static heating for 3.5 h. The obtained AgNWs were washed to remove residual impurities. The final precipitate was dispersed in deionized water, yielding a uniform AgNW suspension.

### 2.3. Preparation of rGO Dispersion

An appropriate quantity of GO was combined with AA at a concentration of 0.3 wt.% and heated to 45 °C while stirring. Following this, the resulting mixture was centrifuged at 1000 rpm for 30 min to yield the modified GO precipitate. The collected precipitate was dried at 60 °C for 12 h to yield rGO powder. Afterward, the rGO powder was reintroduced into deionized water to prepare a 2 mg/mL dispersion, which was then subjected to ultrasonication for 15 min to ensure uniform distribution. Care was taken to avoid excessive ultrasonication time in order to prevent structural damage to the rGO sheets [31]. Finally, a stable rGO dispersion was obtained.

### 2.4. Preparation of rGO/AgNW/PET TCFs

**Figure 1:**
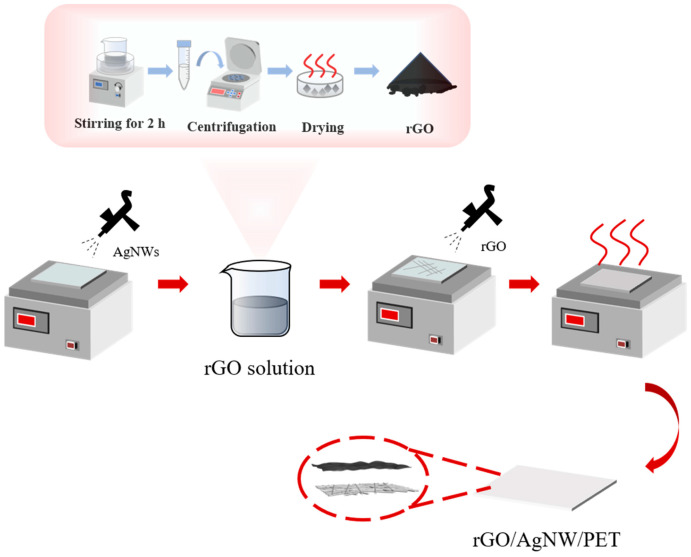
Preparation of rGO/AgNW/PET TCFs.

PET substrates were cleaned by ultrasonication. After cleaning, the substrates were dried for subsequent use. AgNWs synthesized via the polyol method were deposited onto the PET by spray-coating technique to form AgNW/PET films. Considering the susceptibility of AgNWs to oxidation, rGO dispersion was subsequently deposited onto the film surface via spray coating. The two-dimensional rGO nanosheets uniformly covered the AgNW network, forming a protective layer. As a result, rGO/AgNW/PET TCFs were successfully prepared (Figure 1).

### 2.5. Characterization

The chemical compositions of GO and rGO were analyzed using a confocal Raman spectrometer (XploRA PLUS, Horiba, Kyoto, Japan), Fourier-transform infrared spectroscopy (FTIR, Nicolet iS50, Thermo Fisher Scientific, Madison, WI, USA), and X-ray photoelectron spectroscopy (XPS, NEXSA, Thermo Fisher, Waltham, MA, USA). The morphology of GO and rGO were analyzed by transmission electron microscopy (TEM, Hitachi H-7650, Tokyo, Japan). The prepared AgNWs were characterized by X-ray diffraction (XRD, D8 ADVANCE, Bruker, Billerica, MA, USA) using Cu Kα radiation (λ = 1.5406 Å). The surface morphology was observed using a field-emission scanning electron microscope (SEM, Regulus 8100, Hitachi, Tokyo, Japan). The surface roughness was measured using an atomic force microscope (AFM, Bruker, San Jose, CA, USA). The sheet resistance was measured using a four-point probe system (Keithley 2700, Tektronics, Beaverton, OR, USA). The sheet resistance of each sample was measured three times, and the average value was used for analysis. EMI SE was evaluated using a vector network analyzer (Agilent E5071C, Agilent Technologies, Santa Clara, CA, USA). Electrothermal performance was measured by applying a DC (MS-605D, Maisen, Dongguan, China) across the film ends using thermocouples.

## 3. Results and Discussion

### 3.1. Characterization of AgNWs

**Figure 2:**
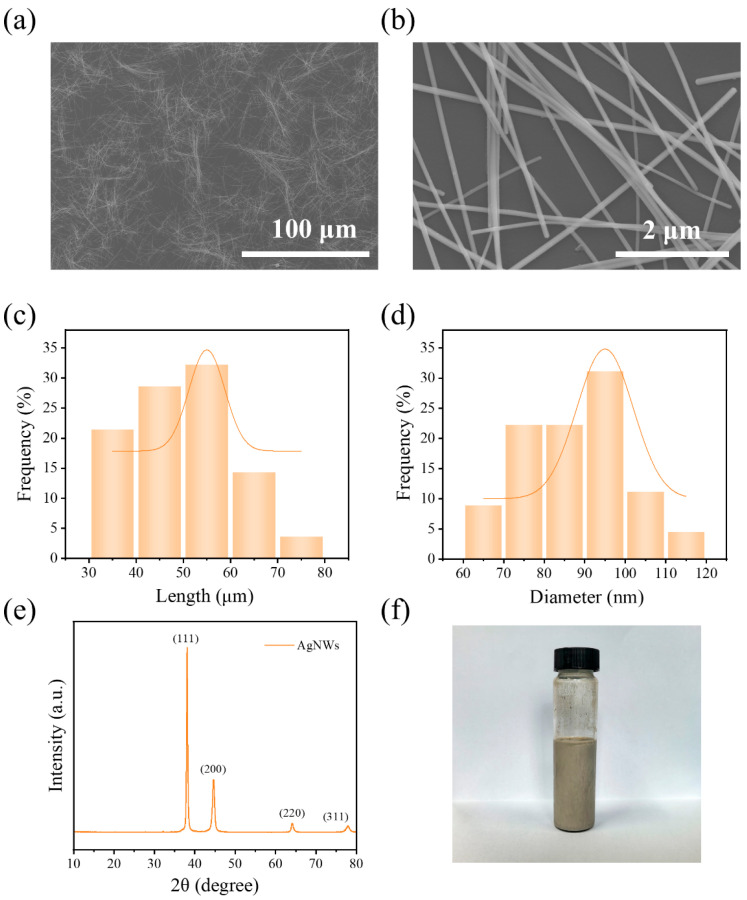
SEM images of AgNWs (**a**,**b**). Length (**c**) and diameter (**d**) distribution histograms of AgNWs, respectively. (**e**) XRD patterns. (**f**) Digital photograph of AgNWs.

Figure 2a,b show SEM images of AgNWs, while Figure 2c,d show their length and diameter distributions. The average length of the AgNWs was found to be 55 μm, and they had an average diameter of 95 nm, which led to a high aspect ratio of about 578. In the XRD pattern (Figure 2e), four distinct diffraction peaks were observed at 2θ values of 38.2°, 44.5°, 64.1°, and 77.8°, corresponding to the (111), (200), (220), and (311) crystal planes of silver, respectively [32]. The lattice parameter was calculated from the XRD peak positions using standard crystallographic relationships for the fcc structure. The obtained value was approximately 4.08 Å, which is consistent with that of bulk silver (4.086 Å), indicating good crystallinity of the AgNWs. Figure 2f shows the photograph of the synthesized AgNWs.

### 3.2. Characterization of GO and rGO

**Figure 3:**
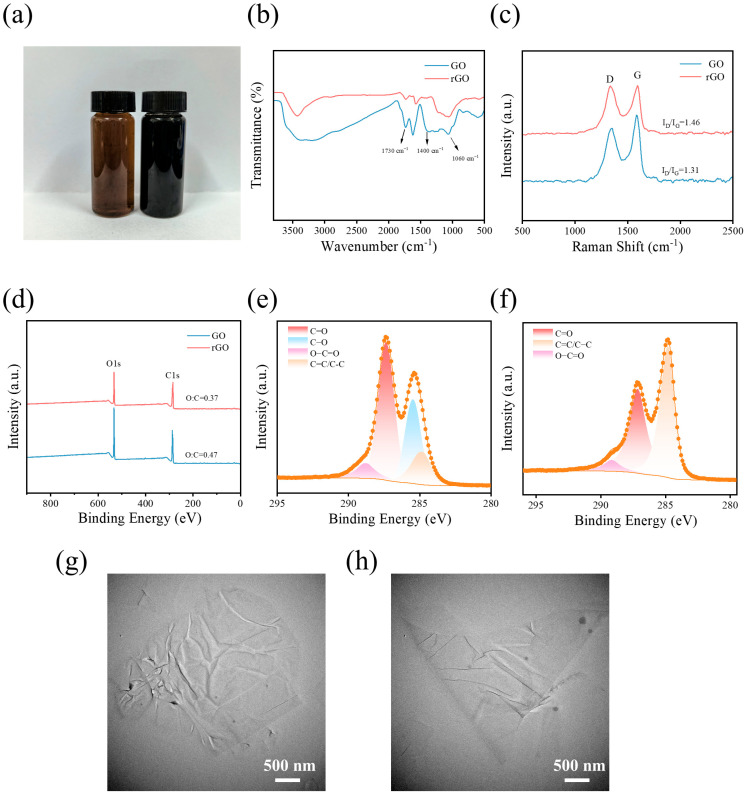
Digital photographs of GO and rGO (**a**), FTIR spectra (**b**), Raman spectra (**c**), XPS spectra (**d**). C1s spectra of GO (**e**) and rGO (**f**). TEM of GO and rGO (**g**,**h**).

Figure 3a presents photographs of GO and rGO. The FTIR spectra of GO and rGO are shown in Figure 3b. Due to its layered structure and abundant hydrophilic groups that contain oxygen, water molecules can easily intercalate between GO sheets, resulting in broad absorption above 3000 cm^−1^. This feature was significantly diminished after reduction to rGO. In the FTIR spectrum of GO, three characteristic absorption peaks were observed at approximately 1060, 1400, and 1730 cm^−1^ [33,34]. The peak at 1060 cm^−1^ is associated with the stretching vibrations of C-O, corresponding to ether (-C-O-C-) and hydroxyl (-OH) groups present on GO. The absorption near 1400 cm^−1^ refers to the bending vibrations of O-H or C-H, particularly prominent when hydroxyl groups exist on the GO surface. The peak at 1730 cm^−1^ is attributed to the stretching vibrations of C=O, typically originating from carboxyl (-COOH) or carbonyl (C=O) groups introduced during oxidation [35]. After chemical reduction with AA, the intensities of these oxygen-containing functional groups in rGO were significantly reduced, indicating the effective removal of -COOH, -OH, and epoxy groups. Raman was further employed to confirm the reduction of GO by AA. Figure 3c displays two prominent peaks for the D and G bands in both GO and rGO. The intensity ratio of the D and G bands (I_D_/I_G_) is utilized as a standard measure to assess the level of graphitization and the concentration of defects in graphene materials [36]. The D band is associated with defects in the sp^2^ carbon framework, whereas the G band reflects the vibrational modes of sp^2^-hybridized carbons [37]. The I_D_/I_G_ value rose from 1.31 to 1.46, suggesting that smaller graphitic structural units were formed during the reduction process. Chemical reduction also led to a significant decrease in residual oxygen-containing groups and partially restored sp^2^-conjugated π-bonds, promoting more ordered stacking of rGO sheets. However, complete restoration of the in-plane C-C σ bonds is still difficult, resulting in reduced graphitic domain sizes [38,39]. The GO and rGO were analyzed by XPS. The O/C ratios for GO and rGO were found to be 0.47 and 0.37, respectively (Figure 3d). That indicates that AA effectively removed a portion of the oxygen-containing groups, confirming the partial restoration of the sp^2^ carbon framework. The C1s spectrum of GO can be resolved into five characteristic peaks, which are assigned to C=C/C-C, C-O (epoxy and alkoxy), C=O (carbonyl), and -COOH (carboxyl) groups, respectively (Figure 3e). In contrast, the C1s spectrum of rGO shows three main peaks (Figure 3f). The C=C/C-C peak intensity increases significantly as the chemical environment changes during reduction. Meanwhile, the peaks corresponding to oxygen-related functional groups are markedly weakened [40]. The C-O groups are preferentially removed during the reduction process, mainly transforming into C=C bonds, with some C-C bonds being formed, suggesting a partial re-establishment of the sp^2^ carbon network. Additionally, the decreased intensity of the -COOH peak further suggests the successful removal of oxygen functional groups. These results confirm that AA acts as an efficient reducing agent for the deoxygenation of GO. TEM was employed to characterize GO and rGO. As observed in the images, GO exhibited a typical wrinkled sheet-like morphology, which is mainly due to the abundant oxygen-containing functional groups, leading to strong electrostatic repulsion between adjacent layers (Figure 3g). After reduction, the sheet structure of rGO became more distinct, with partial restoration of the graphitic domains (Figure 3h). Meanwhile, the degree of wrinkling was reduced, and the surface morphology became relatively smoother. This change indicates that a portion of the oxygen-containing functional groups on GO had been effectively removed during the reduction process, resulting in the partial recovery of the π-conjugated carbon network. The restoration of the structure not only facilitates electron transport within the sheets but also promotes closer inter-sheet contact, resulting in a more continuous conductive network.

### 3.3. Discussion of rGO/AgNW/PET TCFs

**Figure 4:**
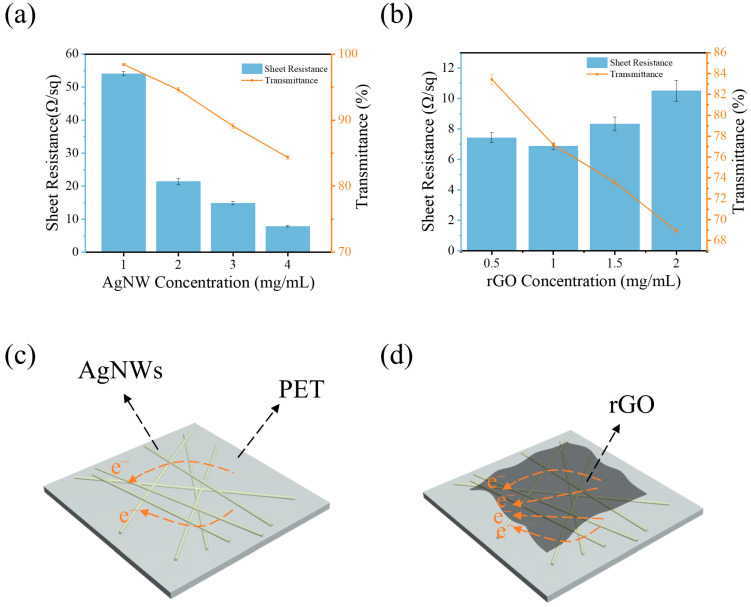
Transmittance and sheet resistance of the film at different AgNW (**a**) and rGO concentrations (**b**). Schematic illustrations of the film before (**c**) and after rGO spray coating (**d**).

On the PET film that had been dried and prepared, different concentrations of AgNW solutions (1–4 mg/mL) were sprayed to investigate the effects of different AgNW concentrations on contact resistance and transmittance. As shown in Figure 4a, with increasing AgNW concentrations, the sheet resistance decreased gradually, while the transmittance exhibited a corresponding decline. To enhance the stability of the films and prevent the oxidation of AgNWs upon exposure to air, rGO was introduced as a protective layer after the initial formation of the AgNW network. Under a constant spray volume, the deposited amount and surface coverage density of rGO on the film gradually increased with increasing rGO concentrations, thereby enabling the investigation of its effects on transmittance and electrical properties. The performance of the TCFs was studied as a function of rGO concentration (0.5–2 mg/mL). After spraying rGO onto the films, the sheet resistance decreased while the transmittance was reduced (Figure 4b). However, as the rGO concentration increased further, the sheet resistance began to increase, whereas the transmittance continued to decrease. This result indicates that an appropriate amount of rGO can form a uniform coating on the AgNWs, offering effective protection while preserving conductive pathways between nanowires, thereby achieving an optimal balance between electrical conductivity and optical transparency. AgNWs, with their high intrinsic conductivity, construct a one-dimensional conductive network that serves as the primary pathway for electron transport. Meanwhile, rGO, possessing a two-dimensional sheet-like structure, can bridge adjacent nanowires and fill the gaps between them, effectively reducing the contact resistance at nanowire junctions. In addition, the rGO sheets improve the continuity and stability of the conductive network, facilitating more efficient electron transport. Consequently, the combination of AgNWs and rGO leads to the formation of a multidimensional conductive network integrating one-dimensional and two-dimensional components, significantly improving the overall electrical performance of the transparent conductive films (Figure 4c,d).

**Figure 5:**
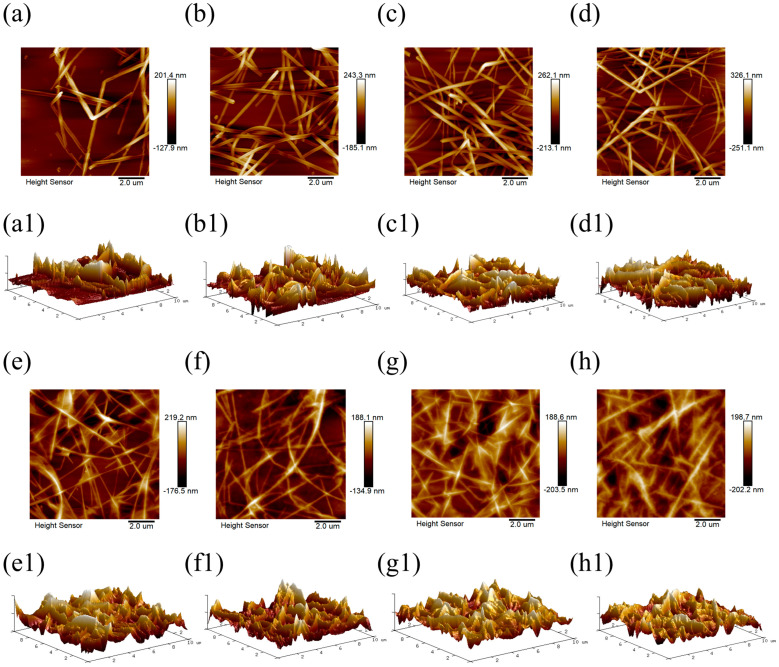
(**a**–**d**) AFM topography images of films prepared with different AgNW concentrations (1–4 mg/mL); (**a1**–**d1**) corresponding three-dimensional AFM images. (**e**–**h**) AFM topography images of films obtained by spraying different rGO concentrations (0.5–2 mg/mL) onto AgNW films with a fixed concentration of 4 mg/mL; (**e1**–**h1**) corresponding three-dimensional AFM images.

In addition, the surface roughness of the TCFs was evaluated. AFM measurements were performed on films prepared with different AgNW concentrations (1–4 mg/mL), as well as on films fabricated by spraying different rGO concentrations (0.5–2 mg/mL) onto AgNW networks with a fixed concentration of 4 mg/mL. The surface roughness was quantitatively analyzed. As shown in Figure 5a–d, the surface roughness gradually increased with increasing AgNW concentration, from 43.8 to 86.1 nm (43.8, 60.8, 69.6, and 86.1 nm, respectively). The corresponding three-dimensional AFM images are shown in Figure 5a1–d1. In contrast, for films with a fixed AgNW concentration of 4 mg/mL, the surface roughness values after introducing different rGO concentrations were 58.0, 45.1, 54.1, and 56.0 nm, respectively (Figure 5e–h). The corresponding three-dimensional AFM images are shown in Figure 5e1–h1. This is mainly due to the effective coverage and filling effects of rGO nanosheets. When rGO coverage is appropriate, it can reduce the contact resistance between nanowires through a bridging effect. However, excessive rGO coverage may lead to the wrapping of nanowire junctions, thereby increasing interfacial contact resistance. Meanwhile, the sheet resistance of the films increases [41]. When the rGO concentration is further increased, excessive accumulation and aggregation of two-dimensional nanosheets lead to localized stacking structures on the film surface, resulting in a slight increase in surface roughness and a deterioration in network uniformity [42]. At the same time, increased rGO loading leads to a thicker surface coating and enhanced sheet stacking, which significantly reduces optical transmittance, limiting its application in optoelectronic devices. Based on these results, TCFs prepared with 4 mg/mL AgNWs and 1 mg/mL rGO were selected as the optimal samples (Rs = 6.8 Ω/sq, T = 77%), ensuring sufficient conductivity while maintaining transparency for subsequent studies, denoted as rGO (1 mg/mL)/AgNW (4 mg/mL)/PET TCFs.

**Figure 6:**
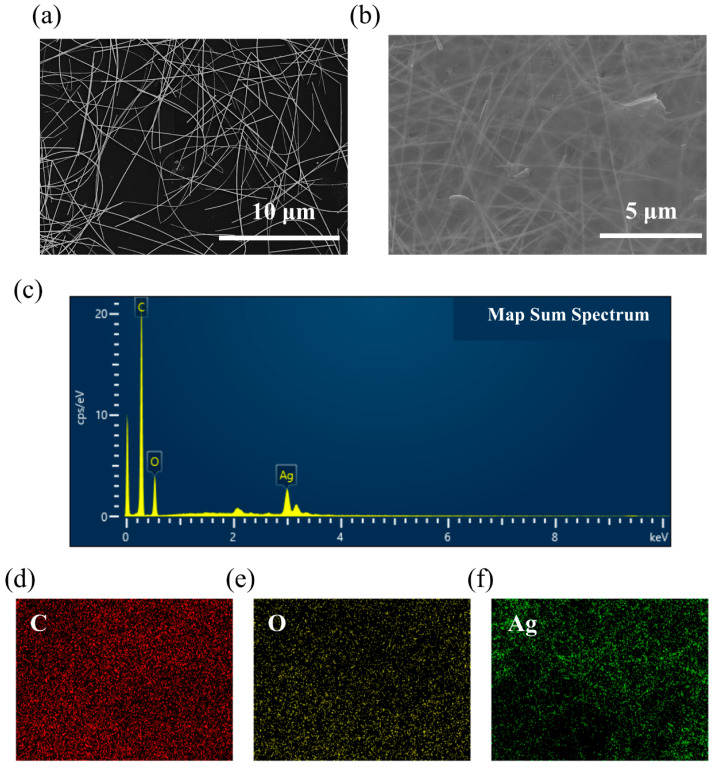
(**a**,**b**) SEM images of the film before and after rGO spray coating, respectively. rGO/AgNW/PET TCFs of (**c**) Map Sum Spectrum; (**d**) C distribution; (**e**) O distribution; (**f**) Ag distribution.

The SEM images of AgNWs before and after rGO spray coating are shown in Figure 6a,b. It can be observed that, after spraying, rGO uniformly covered the AgNWs, forming a continuous protective layer. In contrast, the pristine AgNWs without rGO appeared to be exposed and unprotected. This demonstrates that rGO effectively encapsulated the AgNWs, which can help prevent their oxidation. However, with increasing rGO content, the sheet resistance of the films showed a noticeable increasing trend, suggesting that the continuity of the conductive network was adversely affected. This behavior can be attributed to the fact that excessive rGO sheets may shield or isolate the original AgNWs’ conductive pathways, thereby disrupting the percolation network and ultimately deteriorating the overall electrical performance [43,44]. The elemental composition and spatial distribution of the rGO/AgNW/PET TCFs were characterized by EDS. The EDS spectrum exhibited distinct characteristic peaks of C, O, and Ag, where the C signal mainly originated from the rGO layer and PET substrate, and the O signal was related to the remaining oxygenated functional groups in rGO. The identification of the Ag signal validates the creation of the conductive network made up of AgNWs (Figure 6c). Meanwhile, EDS mapping revealed that C element was uniformly distributed over the film surface, forming a continuous coverage layer. The O element also showed a relatively uniform distribution, whereas the Ag element was mainly localized in discrete regions and was partially covered or surrounded by carbon-rich areas (Figure 6d–f). These results indicate that the rGO layer effectively overlaid and encapsulated the AgNW network, acting as a protective barrier to suppress oxidation and improve the long-term stability of TCFs.

**Figure 7:**
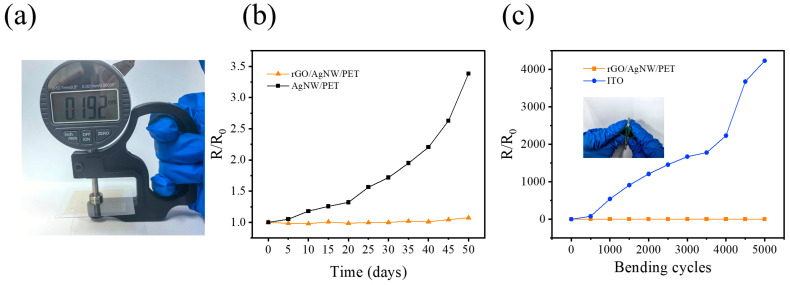
(**a**) The thickness of the rGO/AgNW/PET TCFs. (**b**) Sheet resistance changes of AgNW film and rGO/AgNW/PET TCFs in natural environment. (**c**) Bending cycle tests of ITO and rGO/AgNW/PET TCFs (illustrated with the schematic diagram of the bending test).

The thickness of the films was measured. The thickness of the rGO/AgNW/PET TCFs was 0.192 mm (Figure 7a). To further verify the protective effect of rGO on AgNWs, the films were exposed to air for several days. The air exposure test was conducted under ambient conditions at room temperature. In the absence of rGO, AgNWs are directly exposed to air, resulting in oxidation and a significant decrease in conductivity [45]. In contrast, the rGO/AgNW/PET TCFs, with rGO forming a protective layer over the AgNWs, presented almost no change in sheet resistance (Figure 7b). RGO, acting as a protective overlayer, can effectively hinder the permeation of oxygen and moisture, thereby protecting the AgNWs, suppressing their oxidation process, and improving the stability of the conductive network [46]. Additionally, the mechanical flexibility of the films was assessed through bending cycle tests. The bending test was performed with a bending radius of approximately 2 mm. Compared with ITO films, the rGO/AgNW/PET TCFs exhibited almost unchanged sheet resistance after repeated bending cycles, indicating outstanding flexibility and mechanical durability (Figure 7c).

### 3.4. Electrothermal Performance of the rGO/AgNW/PET TCFs

**Figure 8:**
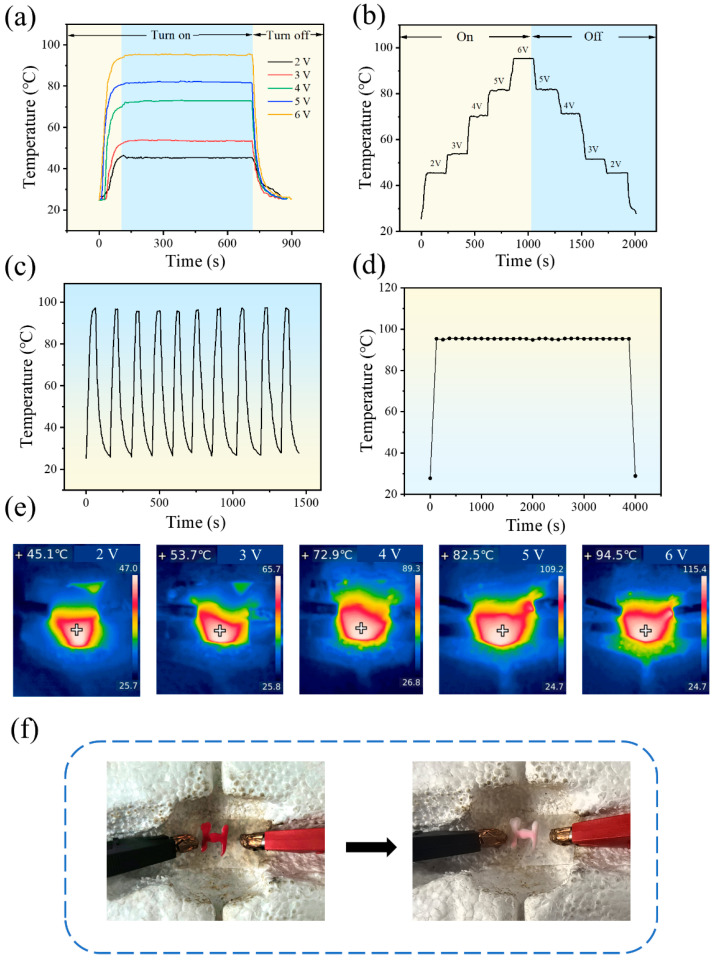
(**a**) Temperature change of rGO/AgNW/PET TCFs at different voltages. (**b**) Temperature variation recorded every 3 min with a voltage increment of 1 V. (**c**) Cycle temperature test under repeated on–off switching at 6 V. (**d**) Stability test under continuous heating at 6 V for 1 h. (**e**) Infrared thermal imaging diagrams at 2 V–6 V. (**f**) Digital photographs of the thermochromic ink test before and after testing.

The electrothermal performance of the films was evaluated. According to Joule’s law, the heating power of the film can be expressed as [47]:

(1) $$P=\frac{U^{2}}{R}$$

where *R* represents the sheet resistance of the film and *U* is the applied voltage, demonstrating that lower sheet resistance TCFs produce more Joule heat. At a specified resistance, the temperature of TCFs increased with increasing applied voltage. The temperature reached a maximum of 95 °C at 6 V, indicating excellent heating performance under low voltage (Figure 8a). Figure 8b shows the temperature evolution of the films under stepwise voltage increments from 2 V to 6 V with 1 V steps, recorded every 3 min. These results indicate that the films exhibited excellent electrothermal performance even at low voltages, with temperatures reaching comparable values under the same applied voltage. To further investigate the stability of the electrothermal behavior, cyclic on–off tests were performed at 6 V. The rGO/AgNW/PET TCFs demonstrated nearly identical heating and cooling profiles over multiple cycles (Figure 8c). Additionally, continuous heating at 6 V for 1 h showed that the films maintained a stable temperature, confirming the good long-term stability of the electrothermal performance (Figure 8d). The rapid electrothermal response arises from the highly conductive AgNWs network, which efficiently converts electrical energy into heat via Joule heating. The introduction of rGO further improved network connectivity and stability, leading to more uniform heat distribution and enhanced cycling stability. Figure 8e presents the infrared thermal images of the film under applied voltages ranging from 2 to 6 V. As the voltage increased, the surface temperature of the film rose significantly. Notably, no obvious local hotspots were observed, indicating excellent thermal uniformity and stability. Meanwhile, the uniformity of the surface heating of the films was evaluated using thermochromic ink with a color-change threshold. The red letter “H” on the ink-coated surface rapidly faded within 20 s, and the color change occurred uniformly across the pattern. This implies that the conductive network was well distributed throughout the film, thereby ensuring uniform heat generation. These results demonstrate the excellent thermal response behavior of the rGO/AgNW/PET TCFs (Figure 8f). A comparison of this work with previously reported electrothermal performances in electrical heating applications is summarized in Table 1.

**Figure 9:**
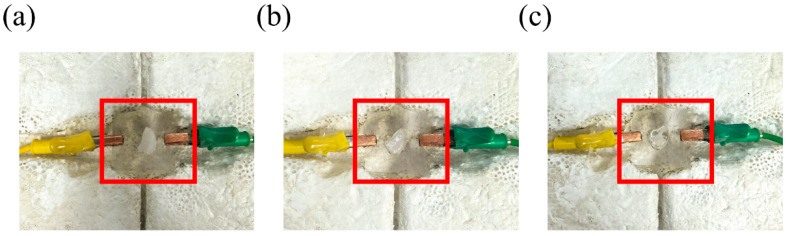
(**a**) The ice cube is placed on the film surface. (**b**) The ice cube begins to melt. (**c**) The ice cube has completely melted.

Benefiting from its rapid thermal response, the rGO/AgNW/PET TCFs exhibited significant advantages in practical applications such as de-icing and defogging, effectively reducing potential safety risks under frost conditions. A small ice cube placed on the film surface began to melt within approximately 15 s (Figure 9a,b) and was completely melted after 30 s of continuous heating (Figure 9c). These results indicate that the film can achieve efficient heat generation and transfer within a very short time, demonstrating excellent electrothermal conversion efficiency and stable heating performance. Moreover, during repeated heating cycles, the film maintained nearly identical de-icing behavior, further confirming its reliability and durability for long-term use. Overall, the rGO/AgNW/PET TCFs showed great potential for applications in de-icing systems, rapid heating devices, and other scenarios requiring instant thermal response.

### 3.5. EMI Shielding Performance of rGO/AgNW/PET TCFs

EMI SE is one of the key parameters for evaluating TCFs, playing a crucial role in determining their reliability and practical applicability. For TCFs, the EMI shielding capability is an important indicator of their functional performance, which is strongly influenced by impedance mismatch. When electromagnetic waves penetrate the shielding material without being reflected, they are gradually reduced in intensity via different mechanisms, including dielectric loss, ohmic loss, as well as dipole and interfacial polarization. The EMI shielding performance was quantitatively evaluated based on the scattering parameters (*S*_11_ and *S*_21_) measured using a vector network analyzer [52]:

(2) $$R={\left|S_{11}\right|}^{2},T={\left|S_{21}\right|}^{2}$$

**Figure 10:**
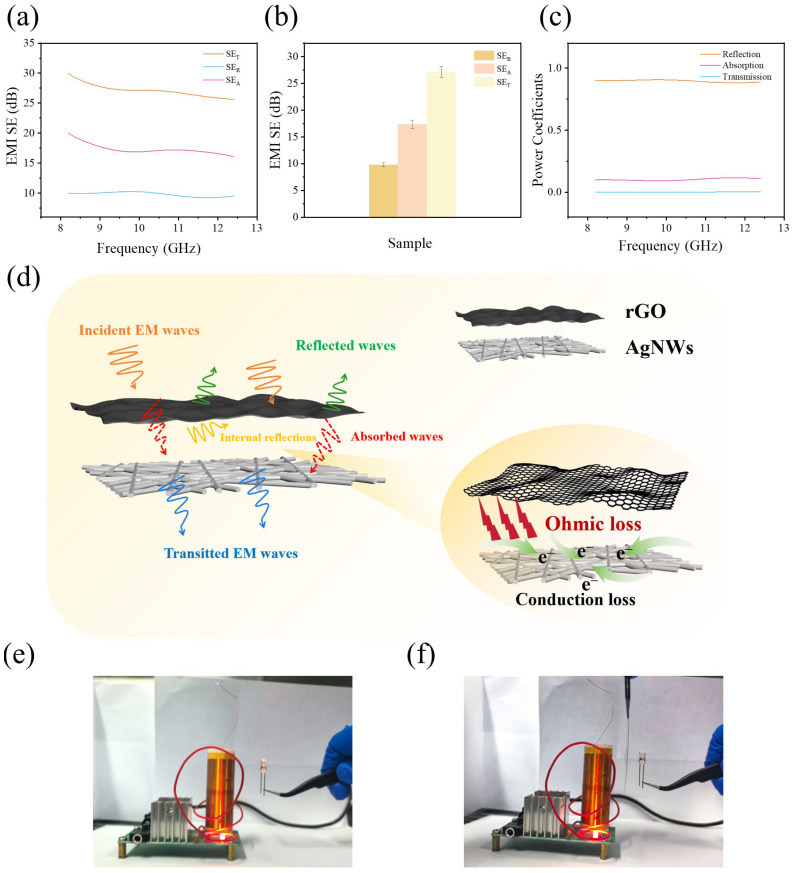
(**a**) The values of SE_R_, SE_A_ and SE_T_ of rGO/AgNW/PET TCFs. (**b**) The average SE_T_, SE_R_ and SE_A_ values of rGO/AgNW/PET TCFs. (**c**) The R, A and T of rGO/AgNW/PET TCFs. (**d**) rGO/AgNW/PET TCFs mechanism diagram. (**e**,**f**) Visualization study on EMI of rGO/AgNW/PET TCFs.

(3) A=1−R−T

(4) $${SE}_{R}=-10log|1-R|,{SE}_{A}=-10log|\frac{T}{1-R}|$$

(5) $${SE}_{T}={SE}_{R}+{SE}_{A}$$

where *R*, *T*, and *A* represent the reflection coefficient, the transmission coefficient, and the absorption coefficient, respectively. *SE_T_*, *SE_R_*, and *SE_A_* correspond to the total shielding efficiency, reflection loss efficiency, and absorption loss efficiency, respectively. EMI shielding performance is primarily governed by the effective conductive network within the composite. Generally, increasing the effective conductive layer’s thickness leads to a more continuous and compact conductive network, enhancing electromagnetic wave reflection and absorption and thus improving EMI SE [53]. However, as the thickness of the conductive layer increases, the optical transmittance of the film decreases significantly [2]. Therefore, the EMI SE of the rGO (1 mg/mL)/AgNW (4 mg/mL)/PET TCFs was calculated to be approximately 27 dB, which is adequate for most practical EMI shielding applications (Figure 10a,b). This level of shielding performance is sufficient to effectively mitigate common electromagnetic interference encountered in daily and industrial environments. The corresponding *A*, *T*, and *R* coefficients are shown in Figure 10c. The mechanism is presented in Figure 10d. When incident electromagnetic waves reach the rGO layer, partial reflection occurs at the interface between air and the rGO layer due to impedance mismatch, while the remaining waves penetrate into the underlying AgNW layer [54]. As the waves propagate through the AgNW network, the interconnected conductive structure induces multiple reflections and scattering, leading to gradual energy dissipation. In addition, abundant free electrons in the AgNW network generate induced currents under the alternating electromagnetic field, resulting in significant ohmic loss and the further attenuation of electromagnetic energy. Meanwhile, the lagged response of interfacial charges to the alternating electric field gives rise to polarization relaxation, enhancing dielectric loss and producing pronounced interfacial polarization effects within the film [55]. A synergistic interaction between rGO and AgNWs is achieved, and the film demonstrates favorable EMI shielding performance. The excellent EMI shielding performance of the TCFs was demonstrated using a Tesla coil (Figure 10e,f). As shown in Table 2, a systematic comparison was conducted between the rGO/AgNW/PET TCFs prepared in this work and those reported for previously TCFs. The results indicate that the present films achieved a comparable balance between optical transmittance and EMI SE relative to existing studies.

**Figure 11:**
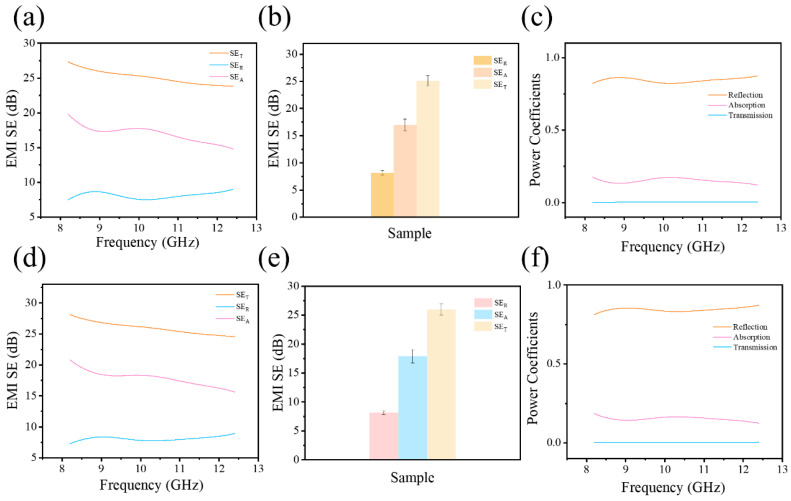
(**a**) The values of SE_R_, SE_A_ and SE_T_ of rGO/AgNW/PET TCFs after bending 5000 times. (**b**) The average SE_T_, SE_R_ and SE_A_ values of rGO/AgNW/PET TCFs after bending 5000 times. (**c**) The R, A and T of rGO/AgNW/PET TCFs after bending 5000 times. (**d**) The values of SE_R_, SE_A_ and SE_T_ of rGO/AgNW/PET TCFs after being stored at 60 °C for 5 days. (**e**) The average SE_T_, SE_R_ and SE_A_ values of rGO/AgNW/PET TCFs after being stored at 60 °C for 5 days. (**f**) The R, A and T of rGO/AgNW/PET TCFs after being stored at 60 °C for 5 days.

The EMI shielding performance under mechanical bending was further evaluated. As shown in Figure 11a,b, the EMI SE slightly decreased from approximately 27 dB to 25 dB after bending, indicating that the composite film maintained good EMI shielding stability under mechanical deformation. This minor reduction can be attributed to subtle rearrangements of the conductive network induced by bending [56]. The corresponding A, T, and R coefficients are shown in Figure 11c. Nevertheless, the well-interconnected AgNW/rGO hybrid network effectively accommodated mechanical strain and preserved continuous conductive pathways, thereby retaining most of the shielding performance after deformation. To evaluate the stability of the EMI shielding performance, an accelerated thermal aging test was conducted by placing the composite films in a drying oven at 60 °C for 5 days. After aging, the EMI SE showed only a slight decrease, remaining at approximately 25.9 dB (Figure 11d,e), indicating good stability under thermal aging conditions. The corresponding A, T, and R coefficients are shown in Figure 11f. This stability is mainly attributed to the rGO layer, which helps suppress the oxidation of AgNWs and maintains the integrity of the conductive network.

## 4. Conclusions

In this study, AgNWs were successfully synthesized via a polyol method, and GO was effectively reduced by AA. A flexible rGO/AgNW/PET TCF was fabricated through a spray-coating process. The obtained films exhibited a low sheet resistance of 6.8 Ω/sq and a high optical transmittance of 77% at 550 nm, demonstrating a favorable balance between electrical conductivity and optical transparency. In addition, the rGO/AgNW/PET films showed excellent electrothermal performance and operational stability. Under an applied voltage of 6 V, the surface temperature rapidly increased to approximately 95 °C and maintained stable heating behavior over time. Meanwhile, the films exhibited an EMI SE of 27 dB, which is sufficient for practical electromagnetic interference suppression applications. Overall, the synergistic integration of rGO and AgNWs resulted in a multifunctional transparent conductive film with combined high conductivity, good transparency, stable electrothermal performance, and effective EMI shielding capability. These results demonstrate the significant potential of the rGO/AgNW/PET films for applications in flexible heaters and electromagnetic shielding devices. The spray-coating technique shows good potential for the scalable fabrication of TCFs.

## Figures and Tables

**Table 1:** Comparison of electric heating performance with previously reported works.

Materials	Steady Temperatures (°C)	Corresponding Voltage (V)	Ref.
GGO/CNT–PET	82.71	15	[23]
UV-curable polymeric resin/AgNWs	93.5	12	[48]
MXene@SWNTs/PC	87.3	6	[49]
PTA/Au dual doped CNT	93.2	10	[50]
AgNW/PVA	73	5	[51]
rGO/AgNW/PET	82	5	This work
rGO/AgNW/PET	95	6	This work

**Table 2:** Comparison of EMI SE with previously reported works.

Materials	Transmittance (%)	Thickness	Frequency (GHz)	SE (dB)	Ref
ITO/Cu mesh	76.1	-	8–12	25.6	[6]
AgNW/CNT@Ag	57.7	-	8.2–12.4	31.42	[56]
Ni-Pd CNT	71.4	100 nm	8–13	21.37	[57]
AgNMs/ePLLA	52.8	20 μm	8.2–12.4	27.6	[58]
graphene/PET	80.5	-	18–26.5	19.14	[59]
rGO/AgNW/PET	77	0.192 mm	8.2–12.4	27	This work

## Data Availability

The original contributions presented in this study are included in the article. Further inquiries can be directed to the corresponding author.
